# *In situ* analysis of dynamic laminar flow extraction using surface-enhanced Raman spectroscopy

**DOI:** 10.1038/srep18698

**Published:** 2015-12-21

**Authors:** Fei Wang, Hua-Lin Wang, Yang Qiu, Yu-Long Chang, Yi-Tao Long

**Affiliations:** 1 State Environmental Protection Key Laboratory of Environmental Risk Assessment and Control on Chemical Process, East China University of Science and Technology, Shanghai, 200237, P. R. China; 2 State Key Laboratory of Bioreactor Engineering & Department of Chemistry, East China University of Science and Technology, Shanghai 200237, China

## Abstract

In this study, we performed micro-scale dynamic laminar flow extraction and site-specific *in situ* chloride concentration measurements. Surface-enhanced Raman spectroscopy was utilized to investigate the diffusion process of chloride ions from an oil phase to a water phase under laminar flow. In contrast to common logic, we used SERS intensity gradients of Rhodamine 6G to quantitatively calculate the concentration of chloride ions at specific positions on a microfluidic chip. By varying the fluid flow rates, we achieved different extraction times and therefore different chloride concentrations at specific positions along the microchannel. SERS spectra from the water phase were recorded at these different positions, and the spatial distribution of the SERS signals was used to map the degree of nanoparticle aggregation. The concentration of chloride ions in the channel could therefore be obtained. We conclude that this method can be used to explore the extraction behaviour and efficiency of some ions or molecules that enhance the SERS intensity in water or oil by inducing nanoparticle aggregation.

Chloride ions, a widespread contaminant in crude oil, are a corrosive substance for oil refineries^1^. To prolong the lifespan of refining equipment, facilities employ a water extraction technique, which exploits solubility differences, to remove chloride ions from the crude oil. However, limited experiments have been conducted to fully investigate chloride extraction in organic solvents. The concentration of chloride ions measured in our targeted crude oil is about 10^−4^ M–10^−5^ M. In the practical production process, hydrocyclone, centrifugal extractor, and other gravity type separators are wildly used for chloride ions extraction. Considering the complexities of the extraction process occurred in the above-mentioned devices, many factors and operation conditions that may affect chloride diffusion have not yet been well characterized. In this case, herein, we study the laminar flow extraction as a basic mass transfer model to provide some technical guidance for related separators.

Laminar flow extraction on a microfluidic chip has been utilized in various fields of study, such as complicated separations, chemical operations, drug detection, and chemical biology^2^,^3^,^4^,^5^, due to its numerous advantages, including fluid control, separation, detection sensitivity, and efficiency^6^. A laminar flow system not only significantly reduces the required sample volume but also facilitates single variable investigations^7^,^8^. Water extraction conducted in microfluidic chips is practical and readily achievable, as the different positions along the microfluidic chip are related to the water-oil contact time at a certain flow rate. Hence, the entire extraction process can be monitored dynamically by measuring the chloride concentration at different positions along the microchannel.

Surface-enhanced Raman spectroscopy (SERS) was first used in 1973 for the analysis of pyridine adsorbed onto a roughened silver electrode^9^,^10^. Throughout the last two decades, SERS has been employed as an ultra-sensitive ultra-rapid analysis technique in the fields of environmental monitoring as well as biological and chemical detection^11^,^12^,^13^,^14^,^15^. The lower limit of SERS for concentration detection can be on the order of magnitude of a single molecule due to the enhancement of the electromagnetic field induced by the resonance of plasmon on nanostructured metallic surfaces^16^. Therefore, the performance of the active substrate is a critical factor in SERS detection. In recent years, silver colloids prepared from the citrate reduction of Ag^+^ have emerged as one of the most popular SERS-active substrates and have been used for diverse applications^17^,^18^,^19^. Chloride ions have been most commonly employed as an aggregating agent to achieve optimal SERS enhancement^20^ for the citrate reduced Ag nanoparticles are negatively charged and chloride ions directly controls the enhancement of the SERS signal from an target molecule through the intense electric field gradients created at nanoparticle junctions which can lead to ‘hot spots’. The concentration of chloride ions will influence the number of ‘hot spots’^22^. Many studies have discussed the changes in the characteristic peak intensity induced by the different degrees of aggregation of colloids by adding a single or a multi-component aggregating agent into the silver colloid^21^,^22^,^23^. However, studies have not yet explored how the changes in SERS intensity can be exploited to quantitatively deduce the concentration of the aggregating agent.

In this paper, we studied the diffusion of chloride ions from an oil phase to a water phase in a microfluidic system using SERS to measure *in situ* site-specific chloride concentrations. First, octadecylsilane (ODS) was used to selectively modify the microchannel to achieve a stable laminar flow and a flat water-oil interface^24^. Sodium chloride dissolved in chloroform, representing the contaminated crude oil, was then pumped into the modified microchannel. A silver colloid solution containing Rhodamine 6G (R6G), representing the water phase, was also injected into the microchannel. R6G was chosen as the target molecule to evaluate SERS performance due to the extensive body of literature that exists for this analyte^25^,^26^,^27^. Finally, *in situ* SERS spectra of the R6G were obtained at different locations in the laminar flow system, and the diffusion dynamics of chloride were investigated based on the SERS detection. Additionally, a numerical model was developed to illustrate the mass transport processes involved in the water extraction process.

## Results

### Detection mechanism

**Figure 1:**
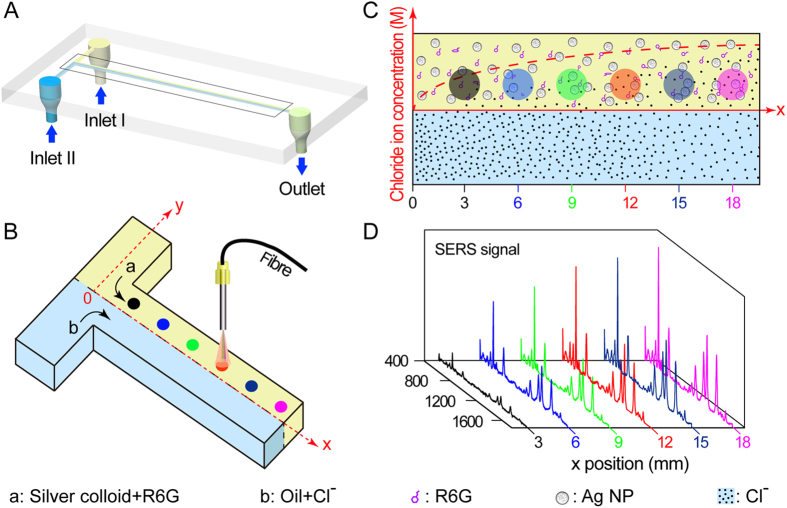
Schematic of a microfluidic chip for chloride ions detection using surface enhanced Raman spectroscopy.

Figure 1 shows a schematic of the microfluidic chip employed for chloride ions detection using SERS. A R6G-containing silver colloid (detailed preparation process and *representative SEM image* are listed in Text S1 and Figure S1) solution and a sodium chloride-containing chloroform solution were pumped into the glass microchannel. Stable laminar flow of the water and oil phases was achieved. Driven by the different affinities of chloride to water and chloroform as well as by the chloride concentration gradient, chloride ions were extracted from the chloroform and diffused into the water phase. Both the chloride concentration and the degree of silver particle aggregation in the silver colloid solution varied along the x axis of the microchannel. Due to these spatial dependencies, the Raman signals were also dependent upon the x-position. SERS spectra at different x positions were recorded using a small portable Raman spectrometer equipped with a bifurcated fibre probe, allowing quantitative analysis of the concentration of chloride ions at different locations within the microchannel. It should be noted that because silver nanoparticles accumulated in the channel due to aggregation or adhesion on the internal channel walls, a new clean device was used for each experiment.

### The effect of the oil phase on SERS analysis

**Figure 2:**
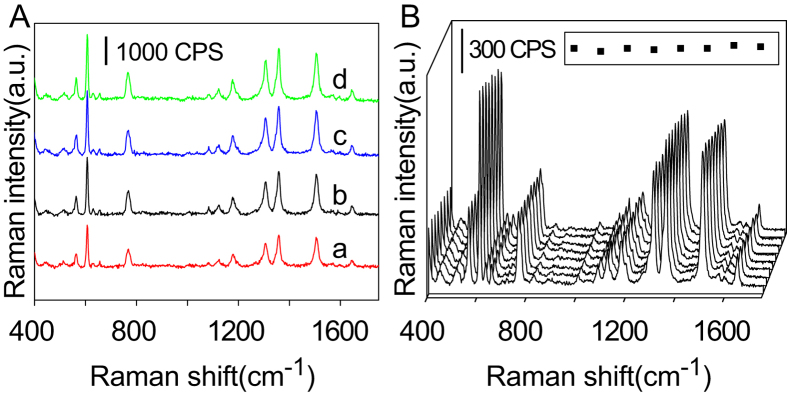
(**A**) SERS spectra of R6G collected from (a) a microchannel perfused with silver colloid mixed with sodium chloride, (b) a microchannel perfused with silver colloid (water phase) and a sodium chloride-containing chloroform solution (oil phase), (c) sodium chloride-containing chloroform solution in a dual-perfused modified microchannel, (d) sodium chloride-containing chloroform solution in a dual-perfused unmodified microchannel. (**B**) SERS spectra of R6G collected from the silver colloid phase at eight different x positions along the dual-perfused microchip at a distance of 10 μm from the interface.

In this study, chloroform, as the oil phase, served as the source of chloride ions (shown in curves a and b of Fig. 2A). Chloroform is a readily oxidizable reagent that produces hydrochloric acid in the presence of oxygen and light^28^. Hydrochloric acid is also an aggregating agent that can diffuse into silver colloids to enhance their SERS spectra. Thus, we investigated the effects of chloroform on SERS detection. Our results showed that when the SERS signal of R6G was collected from the silver colloid phase at eight different x positions along the dual-perfused microchip at a distance of 10 μm from the interface, the signal intensity was virtually identical for all the measurements (Fig. 2B). The standard deviation for the eight SERS signal intensity (1508 cm^−1^) measurements was less than 5%. Thus, chloroform is a suitable oil phase solvent in dynamic chloride extraction and SERS-mediated chloride detection. Meanwhile, it demonstrated that tiny bit of dissolved R6G in chloroform does not affect the detection. The extremely low content of chloride ions produced by chloroform oxidization was insufficient to induce silver colloid aggregation.

### The effect of ODS modification on SERS analysis

Even trace amounts of contaminants lower the accuracy of SERS detection^29^. Consequently, we investigated the effects of ODS modification on SERS analysis of the modified microchannel surface. Our results indicated that the SERS intensities of the representative peaks of R6G were not significantly changed before and after modification (curves c and d of Fig. 2A). Thus, the modified microchannel was deemed suitable for use in the detection experiments presented in this study.

### Construction of a calibration curve of Raman intensity vs. chloride concentration

**Figure 3:**
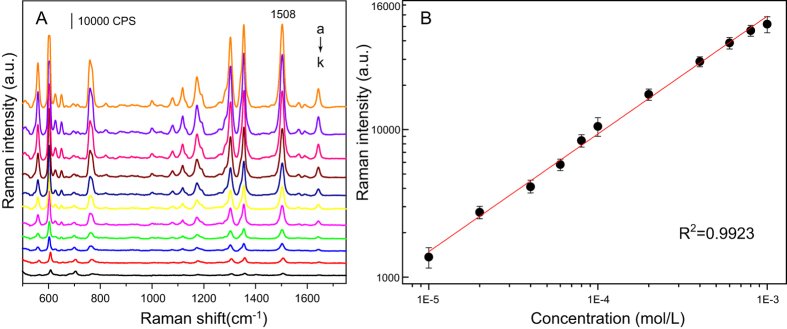
(**A**) SERS spectra of 2 × 10^−5^ M R6G collected from a microchannel containing a silver colloid solution with eleven different chloride concentrations: (a) 10^−3^ M, (b) 8 × 10^−4^ M, (c) 6 × 10^−4^ M, (d) 4 × 10^−4^ M, (e) 2 × 10^−4^ M, (f) 10^−4^ M, (g) 8 × 10^−5^ M, (h) 6 × 10^−5^ M, (i) 4 × 10^−5^M, (j) 2 × 10^−5^, M and (k) 10^−5^ M. (**B**) Calibration curve of Raman intensity vs. chloride concentration. Data points represent the mean value of three replicate samples, and error bars represent the corresponding standard deviation.

To quantitatively determine the chloride concentration, SERS spectra of 2 × 10^−5^ M R6G were recorded for eleven different chloride concentrations at the confluence of the microchannel (where x = 0) (Fig. 3A). Appropriate concentrations of silver colloid, R6G, and sodium chloride solutions were prepared and injected into the microchannel by syringe pumps for accurate test results. Based on the peak intensity of the Raman band approximately 1508 cm^−1^ (C=C stretching), we then plotted a calibration curve of Raman intensity vs. chloride concentration (Fig. 3B). The log-log plot showed an explicit linear relationship (R^2^ = 0.9923), which agreed with previous SERS analysis^30^. This calibration curve was used in subsequent experiments to measure chloride concentrations on the glass microchip.

### Chloride ions detection with SERS

**Figure 4:**
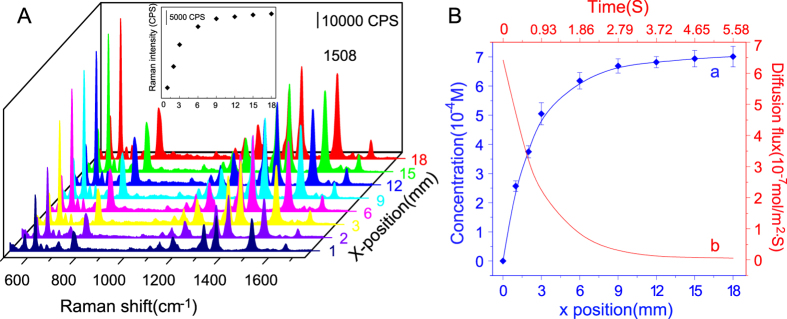
(**A**) SERS spectra of 2 × 10^−5^ M R6G collected from the silver colloid phase at eight different x positions along the dual-perfused microchip at a distance of 10 μm from the interface. (**B**) (a) Chloride concentrations at different x positions along the microchannel; (b) Chloride diffusion flux at different diffusion times. Data points represent the mean value of three replicate samples, and error bars represent the corresponding standard deviation.

The SERS intensities of the various characteristic peaks were closely related to both the number of target molecules adsorbed on the nanoparticles and the degree of silver nanoparticle aggregation. When a certain amount of target molecules is adsorbed onto silver nanoparticles, the SERS signal critically depends on the hotspots that arise from the aggregation of two silver nanoparticles. To evaluate the degree of silver nanoparticle aggregation produced by chloride ions and to measure the concentration of chloride ions accurately, we obtained the SERS spectra of 2 × 10^−5^ M R6G collected at eight different x positions along the side of the microchannel injected with silver colloid. In the inset of Fig. 4A, the Raman intensity at 1508 cm^−1^ is plotted against the x position. The results indicate that the increase in the Raman signal intensity was sharp in the early stages of oil-water laminar flow extraction, but it gradually plateaued as the extraction process continued along the microchannel. From these Raman intensity measurements, we determined the chloride concentrations at different positions in the microchannel (Fig. 4B) by using our calibration curve (Fig. 3B). The concentration of chloride ions increased sharply at the beginning of the dynamic extraction process due to the large concentration gradient of chloride ions. The large concentration gradient drove the chloride ions in the oil phase to rapidly spread to the water phase at the confluence point, where steady laminar extraction began. With two parallel phases flowing in the microchannel, the chloride ions in the oil phase diffused dynamically into the water phase until equilibrium was achieved.

### Numerical Simulation

**Figure 5:**
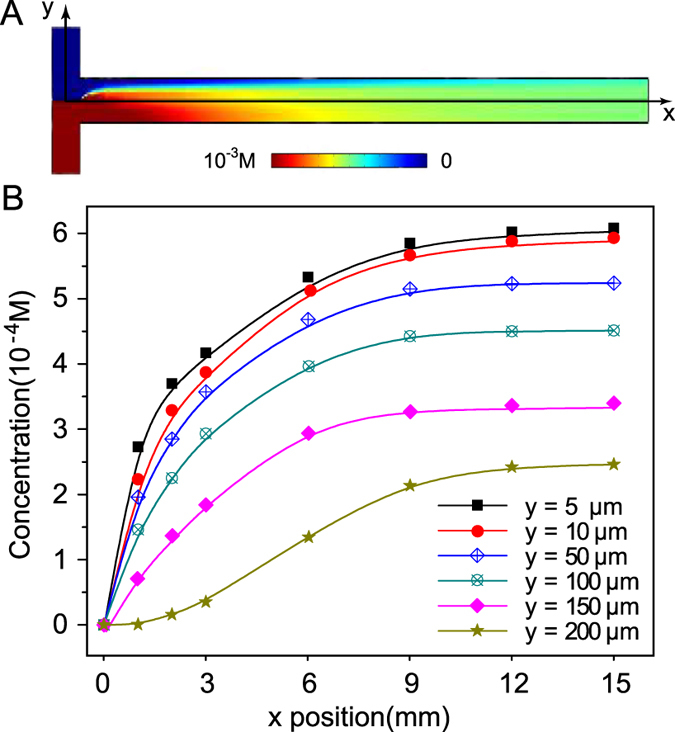
(**A**) Numerical simulation of the scale of transport in the microchannel. Colour map: chloride concentration in the laminar flow device. (**B**) Numerical simulation curves of chloride concentrations in the silver colloid solution at different x positions and different distances from the oil-water interface.

A numerical simulation was performed using COMSOL V4.3a (COMSOL, Stockholm, Sweden) to detect the fluid flow and the exact concentration of chloride ions instead of the laminar microfluidic diffusion diluter^31^. Fluid motion and chloride ions diffusion in the microchannel can be simulated using the steady two-dimensional (2-D) incompressible Navier-Stokes equations with the shallow channel approximation^32^:


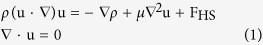



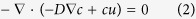


where *u* is the depth-averaged velocity (m/s), *μ* is the dynamic viscosity (Pa·s), *ρ* is the fluid density (kg/m^3^), *D* is the diffusion coefficient of the chloride ions (m^2^/s), and *c* is the chloride concentration (mol/m^3^). The Hele-Shaw force term, 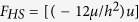, accounts for the resistance to flow due to the out of plane walls in the shallow channel approximation. The simulation geometry is based on the design of the microfluidic flow-focusing device used in our experiments. The fluid and chloride ions concentration fields were estimated by numerical simulation of the above equation with experimentally relevant conditions. For unidirectional flow with a low Reynolds number (*Re* = (*ρuL*/*μ*) ≈ 10^−2^, where *L* is the length scale), lateral mass transport occurs primarily through diffusion. The computed steady state concentration of chloride ions in the laminar flow device is shown in Fig. 5A, and the concentrations of chloride ions at different x positions were recorded. Figure 5B shows that the modelled changes in chloride concentration in the silver colloid solution were similar to the experimental test results. At the beginning of the dynamic extraction process, the chloride concentration increased sharply; subsequently, as the two phases flowed into the microchannel, the chloride ions spread from the oil phase to the water phase, causing the chloride concentration in the water phase to increase gradually and eventually reach a dynamic balance. We obtained similar chloride concentration profiles at distances of 10, 50, 100 and 150 μm from the oil-water interface. At a distance of 200 μm from the interface, the chloride concentration increased slowly at the early stages and but eventually also reached a dynamic balance.

### Diffusion dynamics of chloride ions

Laminar flow extraction is based on the diffusion of molecules across an oil-water interface transverse to the flow direction. This one-dimensional diffusion can be described by the following equation:


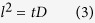


where *D* is the diffusion coefficient (m^2^/s), *t* is the diffusion time or extraction time (s), and *l* is the diffusion distance (μm). In this work, the diffusion distance is 10 μm, and the diffusion coefficient of chloride ions is 2.5 × 10^−9^ m^2^/s. Equation (3) reveals that it takes approximately four seconds for chloride extraction from the oil phase to the detection point located at a distance of 10 μm from the interface. The inlet flow rates of the two streams were 6000 μL/h, which is equivalent to an average velocity of 3.2 mm/s. According to the speed calculation formula 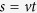, the chloride ions from the oil phase are detected at the position x = 12.8 mm. In the curve of Fig. 4B, the chloride concentration at the position x = 12.8 mm reached a stable state. Experimental SERS results were remarkably consistent with the results calculated by the diffusion dynamics formula.

For laminar flow extraction conditions, the concentrations of all of the components varied only with the diffusion distance *x*. This steady-state diffusion can be described by Fick’s law:


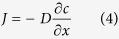


where *J* is the diffusive molar flux (mol/m^2^·s), *D* is the diffusion coefficient (m^2^/s), and 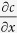 is the concentration gradient (mol/m^4^) acquired by fitting curve *a* in Fig. 5B. Equation (4) was used to plot the rate change of diffusive molar flux (curve b in Fig. 4B). The diffusive molar flux drastically decreased from 6.4 × 10^−7^ mol/m^2^·s to 5.8 × 10^−8^ mol/m^2^·s and gradually approached zero within three seconds. Thus, approximately 91% of the chloride ions were extracted from the chloroform within two seconds, and the extraction was completed when the diffusion mass flux dropped to zero.

## Discussion

In this work, we have demonstrated the ability to combine SERS with microfluidics to quantitatively analyse the diffusion dynamics of chloride ions with site specificity. This system can accurately detect changes in chloride ions concentration on an extremely short time scale (within 1 s) by adjusting the flow velocity and detection position. Our results indicate that SERS can be used to explore the extraction behaviour and efficiency of some ions or molecules that enhance the SERS intensity in water or oil by inducing nanoparticle aggregation. SERS is therefore an ideal technique for the study of extraction processes for industrial applications.

## Methods

### Materials

Silver nitrate (99%), sodium citrate (99%), octadecylsilane (ODS), Rhodamine 6G (R6G), sodium chloride (>99%), and methylbenzene were purchased from Sigma-Aldrich (St. Louis, MO, USA). All other reagents were of analytical grade and were purchased from the Aladdin Reagent Co., Ltd. (Shanghai, China). All solutions were prepared with 18 MΩ·cm deionized water acquired from a Milli-Q Water System (Billerica, MA, USA).

### Apparatus

Raman spectra were recorded with a small portable Raman spectrometer (BWS415, B&W Tek Inc., USA) using an excitation wavelength of 785 nm, a resolution of 5 cm^−1^, and a beam diameter of 10 μm. The Raman spectrometer was equipped with a 1.5 m bifurcated fibre probe and a thermoelectric cooled detector, which facilitated SERS detection with high detection sensitivity. Fluids were driven by two syringe pumps (Longer pump, LSP01-2A, China). Direct observation of the laminar flow was performed using a XSP-8CA biological microscope (Shanghai Optic Instrument). Two glass syringes (5 mL) coupled with PTFE plungers were purchased from the Gao Ke Industry and Trading Co., Ltd (Shanghai, China).

### Glass chip fabrication and modification

**Figure 6:**
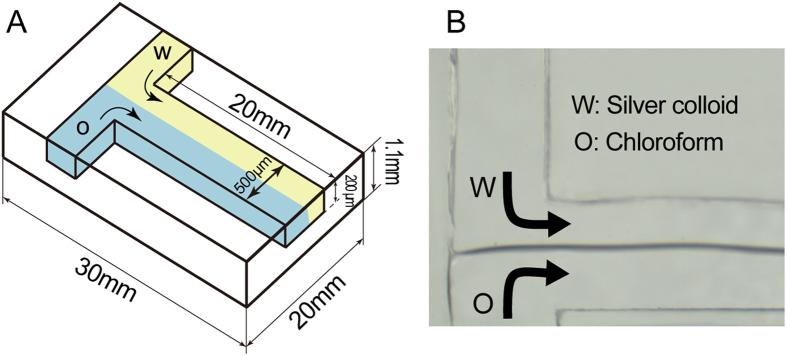
(**A**) Schematic diagram of the glass chip. Colour legend: yellow, hydrophilic pristine region, and blue, modified hydrophobic region. (**B**) Image of laminar flow formed by a silver colloid solution and a chloroform solution.

The chip was composed of two pieces of quartz glass plate that were combined by a direct bonding technique. The dimensions of each plate were 25 × 30 × 1.1 mm. The microchannel was formed on the bottom plate by dry etching^33^. The microchannel cross-section had a width of 500 μm and a depth of 200 μm, and the glass chip had a solvent extraction region with a length of 20 mm. A schematic of the glass chip and microchannel is shown in Fig. 6A.

Hydrophobic modification of one side of the microchannel was achieved by the following procedure^24^,^34^. First, the microchannel was submerged in Piranha solution for one hour. Then, streams of toluene and toluene solution with ODS (1%) were pumped into the microchannel simultaneously for 30 min, with the two streams flowing in parallel. The side of the channel that contacted the ODS solution was thus modified with hydrophobic functional groups, while the other pristine side of the microchannel remained hydrophilic. The toluene stream was terminated after the modification procedure to ensure that the modified area would not be further altered. Finally, the microchannel was washed with ethyl alcohol followed by Milli-Q water for three minutes each. The modified microchannel could maintain a stable water-oil interface, as shown in Fig. 6B.

## Additional Information

**How to cite this article**: Wang, F. *et al.* *In situ* analysis of dynamic laminar flow extraction using surface-enhanced Raman spectroscopy. *Sci. Rep.* **5**, 18698; doi: 10.1038/srep18698 (2015).

## Supplementary Material

Supplementary Information
