# Supplementary material for: In situ analysis of dynamic laminar flow extraction using surface-enhanced Raman spectroscopy
Source: Sci Rep. 2015 Dec 21;5:18698. doi: 10.1038/srep18698 (PMC4685451; doi:10.1038/srep18698)
Supplement: Supplementary Information [file srep18698-s1.pdf]

Supplementary Information

In situ analysis of dynamic laminar flow extraction using surface-enhanced Raman spectroscopy

Fei Wang[1], Hua-Lin Wang[1*] Yang Qiu[1], Yu-Long Chang[1], & Yi-Tao Long[2]

[1]State Environmental Protection Key Laboratory of Environmental Risk Assessment and Control on Chemical Process, East China University of Science and Technology, Shanghai, 200237, P. R. China

[2]State Key Laboratory of Bioreactor Engineering & Department of Chemistry, East China University of Science and Technology, Shanghai 200237, China.

* Corresponding authors

H.L.W.: E-mail address: wanghl@ecust.edu.cn, Tel: +86-21-6425 2748, Fax: +86-21-6425 1894

Supplementary text S1.

Preparation of SERS-Active Silver Colloids. Silver colloids were prepared according to the Lee's method. Briefly, 19 mg of $AgNO_3$ was added into 100 mL deionized water at room temperature and rapidly heated to be boiling, then 2 mL of 1.00 wt. % trisodium citrate solution and 0.02 wt. % polyvinylpyrrolidone was added. The PVP herein was used for stabilizing silver nanoparticles in the solution. The solution was held at boiling for 30 min and cooled to the room temperature. Thus, 60 mL silver colloids were obtained with a concentration about 1 mM. The resulting silver colloids were centrifuged at 6000 rpm for 10 min to isolate AgNPs from excess PVP and resuspended in 100 mL deionized water for the SERS measurement.

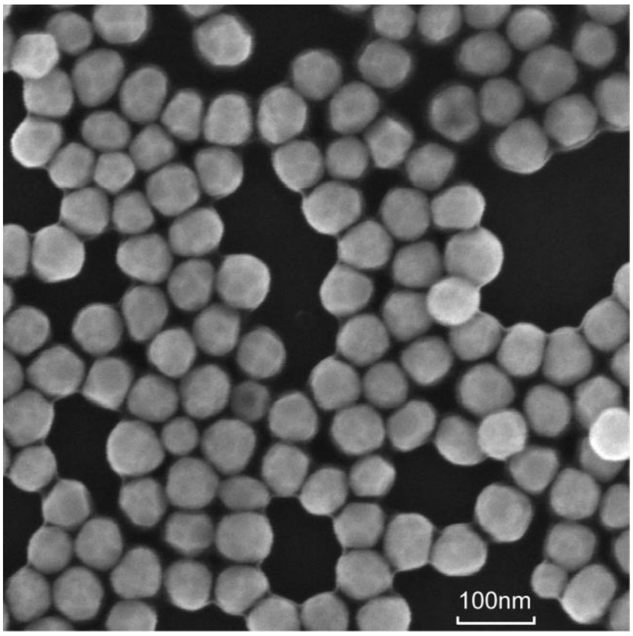

Supplementary Figure S1. *The representative SEM image of AgNPs in the silver colloids*
